# Embed Multisectoral Governance Mechanisms in the Pandemic Instrument for One Health Action

**DOI:** 10.1017/jme.2023.30

**Published:** 2022

**Authors:** Michèle Palkovits, Susan Rogers Van Katwyk, Steven J. Hoffman

**Affiliations:** 1: YORK UNIVERSITY, TORONTO, ONTARIO, CANADA

**Keywords:** Pandemic Instrument, One Health, Global Health Law, Multisectoral Coordination

## Abstract

Despite recognition of the health threat posed at the human-animal-environment interface long ago, One Health has yet to be meaningfully integrated into global pandemic prevention, preparedness, and response. With the negotiation of the forthcoming pandemic instrument under the auspices of the World Health Organization (WHO) — which is inherently restricted by its own constitutional mandate of human health — One Health risks being sidelined once again. Genuine integration of a One Health approach into this treaty will require the institutionalization of formal One Health coordination mechanisms.

Motivated by the collective failures of the COVID-19 response, the world is negotiating a pandemic instrument to govern health in the global arena. This new legal instrument sets out to remedy the inadequate global health architecture at the core of these failures.^1^ While we cannot predict with certainty the source of the next pandemic, significant overlap between strategies needed to mitigate pandemics of various sources means this instrument has the potential to unlock important synergies.^2^ This is particularly true at the human-animal-environment interface, where growing overlap is both heightening risks of zoonotic spillover^3^ and increasing the abundance of resistant genes^4^ — two major sources of (re)emergent diseases.^5^ Recognition of the hazards posed by this interface led to the coining of the term “One Health” in the Manhattan Principles nearly two decades ago.^6^ The many normative commitments that have since followed all stress the importance that the lens of inherent interconnectedness embodied by One Health be central to any framework that attempts to thwart infectious threats to health, no matter their source.^7^

The promise of integrating One Health principles and approaches in this forthcoming pandemic instrument has been described elsewhere^8^ and member states declared their support for the integration of provisions to bring about better One Health coordination.^9^ So far, attempts to integrate One Health approaches into pandemic prevention, preparedness, and response have been confined to soft forms of global health governance.^10^ One Health has yet to be meaningfully integrated into pandemic-relevant treaties, or associated pandemic-relevant plans,^11^ with demonstrable consequences to the coherence of the COVID-19 response^12^ — which remains plagued by an undue focus on response, to the detriment of holistic and deep preventive action.^13^

By their very nature, One Health challenges are intersectoral; spanning the mandates of multiple institutions, One Health challenges are governed by a regime complex of overlapping institutions and authorities.^14^ As such, while the World Health Organization (WHO) will host the instrument, whether it is in fact the ideal forum is contested^15^ as it is inherently restricted by its own constitutional mandate of *human* health.^16^ Consequently, the focus of any instrument negotiated under its auspices is apt to regress to the institution’s sectoral specialization, and actors operating outside human health may be vulnerable to sidelining and under-resourcing. For this reason, genuine integration of a One Health approach into a pandemic instrument overseen by the WHO will require the institutionalization of formal One Health coordination mechanisms.^17^

As the intergovernmental negotiating body drafts the new pandemic instrument,^18^ there is an opportunity to establish smarter global governance arrangements that not only promote but also mandate global intersectoral and interinstitutional equity, cooperation and solidarity,^19^ and the One Health perspective vital to the success of pandemic preparedness and response.^20^ With this opportunity comes an urgent need to consider the type of mechanism best suited to this purpose. The following is an exploration of 6 such mechanisms and the possibilities they offer.

As the intergovernmental negotiating body drafts the new pandemic instrument, there is an opportunity to establish smarter global governance arrangements that not only promote but also mandate global intersectoral and interinstitutional equity, cooperation and solidarity, and the One Health perspective vital to the success of pandemic preparedness and response.

## Consideration of Different Mechanisms

Any valuable One Health mechanism established by the new pandemic instrument must foremost facilitate and coordinate multisectoral engagement and cooperation between global institutions. In practice, this means that a One Health mechanism should ensure that actors share data, evidence, information, and recommendations; are reciprocally participating and represented in strategy and planning; are continuously consulting one another; and ultimately, are acting in a concerted effort.^21^ The mandates of these mechanisms should also align with broader pandemic instrument objectives, to contribute to redressing the existing regulatory gap in pandemic prevention, preparedness, and response. Various global functions that must be improved or consolidated to strengthen pandemic governance, and that could fall within the scope of a One Health mechanism have been identified elsewhere, among those are: regulatory obligations around activities and places, integrating and sharing surveillance, bridging the science to policy interface, strengthening monitoring and investigative powers, ensuring compliance and accountability, and enabling support and capacity-building.^22^ Finally, a successful One Health mechanism’s features and design will embody principles of good governance such as equity, legitimacy, credibility, and transparency, both as ideals in themselves, and as means to sustained collaboration^23^ and more effective policy responses.

Global institutions have established several coordination and collaboration mechanisms, of varying purposes and designs. Drawing upon mechanisms that have been instituted towards other multisectoral problems, including climate change, food safety and food security, as well as previous One Health collaboration models,^24^ we explored 6 potential One Health multisectoral engagement mechanisms that could strengthen One Health coordination and engagement (Table 1).^25^ We investigated each mechanism’s potential contribution to pandemic and One Health governance through an assessment of their capacity to coordinate global intersectoral actors, their alignment with broader pandemic instrument objectives and principles of good governance, and the practicalities and limitations of mechanism designs based on lessons learnt from previous models.

**Table 1:** Summary of 6 Possible One Health Mechanisms

Mechanism	Model	Purpose	Merits	Limitations and Design Considerations
Independent One Health Panel on Pandemic Threats	Intergovernmental Panel on Climate Change	Evidence synthesis, assessment, consolidation, and synthesis to bridge the science and policy interface	Strengthens the science-to-policy interface Highly credible due to independence and epistemic authority	No implementation power Legitimacy is threatened by lack of representation and transparency in processes Potential for politicization during endorsement
One Health Standard Setting Commission	Codex Alimentarius Commission	Harmonize international practice through standard, method, and target setting	Enables coherent action Facilitates monitoring, accountability, and capacity building Reference point may improve instrument compliance	Legitimacy is threatened by non-transparent and inequitable representation Scientific credibility undermined by lack of transparency in processes
Intergovernmental One Health Consultation Forum	Committee on World Food Security	International and cross-sectoral coordination and policy convergence	Enables coherent action and shared learning Facilitates monitoring, accountability, and capacity building Inclusive participation improves equity and decision-making Increases decision ownership	Little scientific credibility Eligibility and roles must be designed to ensure inclusivity and equity
Special Rapporteur on One Health	Special Rapporteurs for human rights	Monitoring progress on pandemic instrument obligations	Independence enables stronger transparency and accountability Facilitates capacity building Flexibility allows responsiveness to context and events	Limited implementation or enforcement power Limited capacity due to broad mandate and few resources Little capacity to coordinate global level multisectoral action
UN System One Health Coordinator	UN System Influenza Coordinator	Interagency coordination and national capacity building	Supports capacity building that is responsive to context Facilitates monitoring, accountability Increases decision ownership	Limited capacity due to broad mandate and few resources Little capacity to coordinate global level multisectoral action
Joint Programme on One Health	Joint UN Programme on HIV/AIDS	International and cross-sectoral coordination, policy convergence, harmonised and concerted action, and capacity building	Encompasses features of many alternative mechanisms Can generate sustained political buy-in Stronger interagency linkages and equity	Politically infeasible to establish due to burden to influential countries and mission shrink for UN agencies Decentralized design may increase financial sustainability

Ultimately, we propose that the forthcoming pandemic instrument co-embed two symbiotic mechanisms, which taken together would unlock synergies and build a resilient and holistic One Health coordination architecture at the core of global health governance. To this end, we recommend that the Independent Panel, converging the science, and the Intergovernmental Forum or Standard Setting Commission, converging policy, both be embedded in the instrument.

What follows is a fuller description of these 6 potential mechanisms, their structure, merits, and design considerations, a deeper analysis and discussion of the trade-offs between these mechanisms, and how they led us to our recommendation.

## Six Mechanisms for One Health

### 1. Independent One Health Panel on Pandemic Threats

#### Structure and Contribution

A comprehensive pandemic instrument could launch an authoritative *Independent One Health Panel on Pandemic Threats*,^26^ a permanent multisectoral infrastructure for the science and policy interface.^27^ Drawing on the success of similar initiatives in the environmental context, such as the Intergovernmental Panel on Climate Change (IPCC), this Panel would ensure that the global scientific evidence base on the impact and future risks posed by the range of pandemic threats is regularly assessed and synthesized. By doing so, this mechanism can generate policy-relevant scientific insights into the risks of emerging health threats arising at the human-animal-environment interface, and ultimately enable policy convergence at this interface. The scientific consensus brought forth by the Panel could in turn serve as a basis for normative guidance, standard and target setting, and could feed into the expert-driven work of the One Health High-Level Expert Panel. Proposals for similar structures have been described elsewhere,^28^ some of which have advocated for an even more expansive mandate, which would “allow for policy-prescriptive conclusions and offer technical support in the form of guidelines, capacity building, and other aids to implementation.”^29^

#### Merits

The success of model panels is in part attributable to their scientific authority and credibility, as well as their functional link to treaties, which ensures their outputs are directly considered by convention bodies.^30^ Like the IPCC, the *Independent One Health Panel on Pandemic Threat*’s credibility would be recognized through its independent and scientific foundation.^31^ This joint epistemic authority and commitment to political neutrality also drive policy impact by galvanizing political, civil society and media support.^32^

#### Design Considerations and Limitations

Two key design limitations for this type of mechanism include genuine inclusivity and the risk of politicization of the evidence.^33^ First, the IPCC has been perceived as a hegemonic actor due to its authorship makeup, which has undermined the Panel’s legitimacy in many parts of the world.^34^ Diversity and equitable representation become especially important as Panels “engage[] more closely with policy-driven questions,” where worldviews may introduce valuable disagreement and complexity.^35^ Mandating and funding the inclusion of a diversity of expertise and perspectives from the whole of the scientific community, ensuring global and cross-disciplinary representation, and requiring transparent and equitable authorship and peer-review selection processes can help overcome this challenge.^36^ Moreover, while integrating member states and the UN system in the production of knowledge can improve political salience, political approval of scientific outputs can put the organization’s independence and scientific integrity at risk.^37^ To limit political interests from leaking into the process,^38^ a lighter-handed intergovernmental endorsement may be preferable.

### 2. One Health Standard Setting Commission

#### Structure and Contribution

The pandemic instrument could mandate the creation of a technical governance mechanism to guide and clarify the instrument’s implementation. Analogous to the *Codex Alimentarius Commission*, a *One Health Standard Setting Commission* would harmonize international practice through the establishment of joint standards, a necessity to achieve coherence and facilitate action at the human-animal-environment interface.^39^ This mechanism would bring together the evidence from across sectors to inform the development of a set of common global technical standards, methodologies, and targets on One Health issues.

The Standard Setting Commission’s role could also improve instrument compliance in two main ways. First, this type of work enables and facilitates norm creation and dissemination, and through a direct instrument link, becomes a reference for dispute resolution.^40^ This in turn promotes compliance and clarifies ambiguities regarding certain state obligations.^41^ Second, this body could facilitate the recognition, monitoring, and closing of gaps in core capacities, which are often at the root of non-compliance. Rather than burdening countries, the standards it establishes would become the reference upon which minimum state capabilities are assessed,^42^ and may help countries identify target gaps and secure funds to improve capacity.

#### Merits

Mandating the creation of a Standard Setting Commission through a instrument imbues it and its outputs with greater authority. Although the Codex Commission was established as an informal standard setting organism decades before the World Trade Organisation Agreements came into force, the designation and adoption of the Codex as a global reference point in the global trading system catalyzed a shift in the Codex’s legal status: that which had previously been a voluntary exercise gained a compulsory character and political importance.^43^

#### Design Limitations and Considerations

Some argue the Codex suffers from a democratic deficit: member states lacking the capacity to implement standards are often also unable to participate in standard setting activities or to be Chairs for the same reason.^44^ The underrepresentation of consumer interests and overrepresentation of industry within the observers and national delegations at the Codex has also been flagged.^45^ Requirements for co-chairmanship by underrepresented countries and mandatory training for the role, as well as financial support for participants, could bring about fuller participation by countries and non-governmental organizations alike.^46^

Despite being intended as a technocratic body, the Codex also lacks scientific credibility. Corporate influence has infiltrated the evidence review process: many studies are conducted by industry scientists, and many committees rely on industry expertise.^47^ A transparent process for the assessment of scientific evidence would have to be established to inform a *One Health Standard Setting Commission* in their work.

### 3. Intergovernmental One Health Consultation Forum

#### Structure and Contribution

The pandemic instrument could establish an *Intergovernmental One Health Consultation Forum*, a permanent state-led multistakeholder convening forum modelled after the Committee on World Food Security (CFS). In regular joint sessions which converge diverse actors around a shared table — including member states, relevant UN agencies, civil society, and private sector actors — this forum would serve as a setting for collective engagement on pandemic-relevant issues across sectors. The Forum’s mandate could include coordination of action, shared learning and capacity building, monitoring of progress, and promoting member state accountability.^48^ Bringing together the expertise and guidance from relevant UN agencies, together with high-level and multisectoral government representation, this mechanism has the potential to converge policy agendas, align targets, and synergize action at the global and national levels. This type of multilateral consultation space could additionally provide a setting for dispute resolution between member states.^49^

#### Merits

Others have already recognized the potential of this type of mechanism as a means to fill a gap in the pandemic governance architecture.^50^ Establishing a platform purposefully designed to foster inclusive and meaningful consultation across a broad range of stakeholders enhances multisectoral participation in policymaking, which in turn can bring about better and more equitable decision-making. This structure would also have the added advantage of a direct and systemic interface with member state representatives, thus benefiting from the political tractability and legitimacy of endorsement by the decision-making body, and directly feeding into decision-making processes and increasing ownership.

#### Limitations and Design Considerations

Criticism of the world food system in the wake of the 2008 food crisis led to calls for governance arrangements that would enable better international coordination, triggering two major reforms to the CFS.^51^ First, broad and meaningful inclusivity was unlocked following the revision of participant eligibility and roles, which helped to ensure that decisions were informed by experiences on the ground and resonated across all stakeholders.^52^ Second, the reforms brought about the creation of an expert panel inspired by the IPCC to respond to a need to “improve the way knowledge is conveyed to multi-stakeholder political platforms,” establish a function of “collective learning” and bring about a “common understanding” of food security.^53^

### 4. Special Rapporteur on One Health

#### Structure and Contribution

The pandemic instrument could also create the role of Special Rapporteur on One Health. Much like the UN Human Rights Council’s Special Rapporteurs, a One Health Rapporteur would be mandated to raise awareness, generate support, propose solutions, and report on the progress of priority One Health challenges, from a One Health perspective. Complementing other self-reporting mechanisms for global health progress, the appointed would promote the accountability of member states to the terms of the pandemic instrument by monitoring the activities taken to address global health threats in alignment with One Health ideals, holding inquiries into specific issues, responding to complaints, and generating reports on gaps between targets and reality. The Special Rapporteur could simultaneously support member states’ efforts by offering guidance and support in addressing challenges, advising on the development of global guidance and training, or mobilizing extra financing to support states that are as yet unable to meet specific obligations.^54^

#### Merits

Special Rapporteurs have been hailed as one of the UN human rights system’s most innovative and important mechanisms.^55^ The advantages of adopting this mechanism for the purposes of One Health lie in its flexibility and independence. Given their broad mandates, Special Rapporteurs can be adaptable and responsive to current or urgent events.^56^ Additionally, they are unlike other UN mechanisms or bodies in that they are staunchly independent of the UN system and its political constraints — allowing them a high degree of autonomy in their work, the ability to speak freely and to maintain a reliable sense of the ‘on-the-ground’ reality.^57^ For these reasons, Rapporteurs often become the public face of an issue and may spur significant public support.^58^

#### Limitations and Design Considerations

Despite their visibility and mandate of accountability, in practice, Special Rapporteurs have little power to implement and enforce, as there are no effective follow-up procedures for accountability reports.^59^ Furthermore, in the absence of substantial investigative capacity, they struggle to verify information.^60^ Calls for alternate means to alert the world to potential public health events in the wake of COVID-19 have highlighted the need for a pandemic instrument to enable (1) non-state actor information to be received without state verification, and (2) independent experts to conduct investigative missions.^61^ A Special Rapporteur could theoretically serve this inspection role, though this would require a significant reshaping of the powers that accompany the position.

### 5. UN System One Health Coordinator

#### Structure and Contribution

The pandemic instrument could build upon a previous One Health coordination mechanism for health threats, the *UN System Influenza Coordinator*. In this renewed version of the role, the *UN System One Health Coordinator*’s scope of work would encompass all pandemic threats emerging at the One Health interface. The mandate of the appointed One Health Coordinator would be to enable robust yet flexible cross-sectoral integration at the national, regional, and global levels, and to ensure interagency and multilevel coordination within and outside the UN.^62^ To accomplish this, the Coordinator would strengthen partnerships and communication between stakeholders, prepare a strategic framework for action, assess and advise on UN agency priorities, targets and action, and identify gaps and opportunities for deduplication and synergies, among other tasks.^63^

A major part of a Coordinator’s role is to manage an integrated *Coordination System*, which operates within the broader UN Sustainable Development Group’s (UNSDG) Resident Coordinator (RC) system.^64^ RCs and UN country teams work through the Coordination System to monitor and report on preparedness progress and compliance with international standards, mobilize funds and direct the efficient use of resources, and coordinate national capacity-building programs from external actors.^65^ Altogether, this approach bridges the interface between sectors and scales of pandemic planning and response — ensuring alignment and coordinated action between human-animal-environment actors, technical and non-technical partners, and national and international structures.^66^

#### Merits

The RC system places countries at the centre of pandemic planning, as country offices have the flexibility to determine their own objectives in response to contextual needs. This highly adaptable and targeted support is enabled by avoiding formal arrangements, the RC system preferring instead to work through relationship-building.^67^ This approach has the added benefit of generating strong national ownership of development action — the strong ties between the UN teams and ministries are fortified by principles of inclusivity and consensus in decision-making.^68^

Unlike other mechanisms described herein, this mechanism is especially well-suited to advancing capacity building and improved monitoring of progress, priorities, and expectations of countries with less capacity to implement. In contrast, global norms and standard setting are typically priorities for countries with already strong implementation capacity.^69^

#### Limitations and Design Considerations

Operating within the UNSDG system would entail being constrained by its structural limitations. The flexibility endowed through the absence of formal processes for coordinating development efforts has drawbacks, namely that RCs must rely on their own leadership and persuasiveness to generate collaboration and promote funding, while the UN agencies involved face little requirements or accountability.^70^ Many RCs and country teams also already face significant resource constraints. Countries that require the most support are often those confronted by multiple development challenges. In these complex settings, the RC’s mandate expands and their role becomes much more difficult to manage, all the while under the same resource constraints.^71^

### 6. Joint Programme on One Health

#### Structure and Contribution

Finally, the instrament could establish a new UN partnership to govern One Health issues of a scale comparable only to the Joint UN Programme on HIV/AIDS (UNAIDS). UNAIDS was established due to “the need for a broader-based, expanded response […] and better-coordinated UN system support to countries”^72^ — much as is needed today across One Health issues. A bigger, bolder undertaking than the other mechanisms, a ‘UN-One Health’ could theoretically encompass many of their functions and thus respond to many One Health gaps.

The promise of this approach and a more detailed discussion of considerations for its governance has been described in previous scholarship.^73^ In short, it is governed by a Board composed of a representative group of member states, UN agencies, and NGOs, which guides the Joint Programme and sets an agenda and policies. The Board is supported by a cosponsoring agency body, which ensures reciprocity between the agencies and the programme, and “operationalizes” the Board’s decisions,^74^ including input from the agencies into strategies, and alignment of agencies’ work with the joint programme agenda.^75^ These, along with the many other groups that make up the Joint Programme could be adapted and scaled to best respond to the gaps in the current global health governance architecture.

#### Merits

This type of structure possesses features, and accordingly merits, of many of the previously described mechanisms. The Board at the core of the programme’s governance structure serves, in essence, as an Intergovernmental Forum for high-level consultation and discussion; the programme can establish bodies that work to interpret the evidence to set targets and standards and harmonize action; and through collaboration with the UNDP, it enjoys a comparable arrangement with the Resident Coordinator system to the UN System Coordinator, enabling flexible and responsive national support.^76^

The financial and political requirements for the establishment of such a body are so significant that if successful, it could generate sustained political buy-in. Moreover, it is likely to involve more UN agencies, in a more equitable manner, bringing about greater interdisciplinarity.^77^

#### Design Considerations and Limitations

The demands for the delegation of mandate and authority from both states and agencies make such a proposal politically infeasible. It is unlikely that the realities of this endeavor’s burden and benefit sharing will generate the needed buy-in and leadership from influential countries.^78^ In parallel, the creation of a new and separate agency will be perceived by many UN agencies, particularly those directly engaged in this issue, as a risk to their leadership, mission, and resources.^79^ Moreover, the UNAIDS record suggests that voluntary funding is unreliable in sustaining the budget of such a large body, and eventually falters.^80^ UNAIDS recently underwent an important restructuring process to adapt to unpredictable and inflexible financial commitments, suggesting that a more decentralized, network approach to a UN-One Health may prove a more sustainable design.^81^

## Analysis and Recommendation

Each of the mechanisms described herein could strengthen the pandemic instrament by encouraging and facilitating One Health coordination. Beyond reinforcing cross-sector linkages, these mechanisms could also make pandemic governance more scientifically credible or democratically legitimate, and could contribute to other instrament goals, such as capacity building or monitoring of member state progress.

While the Joint Programme looks to be the most comprehensive choice, it is unclear whether the benefits of a Joint Programme would outweigh the costs of such an endeavour, at least not in a manner unrivalled by alternative mechanisms. A UN-One Health is unlikely to be politically feasible or to be sustainable in the long term; the global political momentum that enabled the launch of UNAIDS is incomparable and has since subsided, with consequent financial fluctuation, cutbacks and restructuring.^82^ Moreover, the troubled launch of UNAIDS casts a long shadow over any similar proposals, and many UN agencies are unlikely to welcome a repeat.^83^

Meanwhile, the Special Rapporteur and the UN System Coordinator could both significantly contribute to instrament implementation and have the advantage of being flexible and relatively autonomous mechanisms. However, both face criticism of having too broad of mandates and too few resources and would lack the necessary capacity to successfully coordinate multisectoral action.^84^ What’s more, while providing valuable national One Health implementation and coordination, neither focuses on strengthening global One Health action, the principal objective of embedding such mechanisms into the instrament.

Considering the political feasibility of mandating the creation of these mechanisms, as well as their potential impact and contribution to the pandemic instrument’s objectives, it is the view of the authors that the pandemic instrament ought to co-embed the *Independent One Health Panel on Pandemic Threats*, together with either the *Intergovernmental Forum* or the *Standard Setting Commission*.

An Independent Panel is plainly needed. Evidence convergence is the necessary first step towards coordinated action, especially on a matter as interdisciplinary as One Health. COVID-19 exposed member states’ proclivity for a discretionary interpretation of the data, evidence and risks, as shown by their regular deviation from WHO recommendations.^85^ An accessible, transparent, and authoritative source of scientific evidence might generate pressure towards policy alignment with the science, if not treaty compliance. Well-designed political participation and endorsement of scientific panel outputs further engenders “unimpeachable authority,” and can influence major legal developments.^86^

Paired with the Independent Panel, the Intergovernmental Forum has the potential to be highly impactful. Drawing on the evidence base generated by the Panel, it would engender policy convergence for all types of action. Offering a space for member state discussion, consultation, and coordination is “essential for managing the political dimension that inevitably characterizes an international crisis,” and can bring about improved effectiveness and greater harmonization. The Forum would bring about greater democratic and political legitimacy, building trust and accountability between actors, shaping consensus, and, ideally, bringing about better and more equitable solutions through meaningful and representative participation.

The scope of the Panel’s contribution to multisectoral coordination is limited to the realms of research and advice, having no implementation power. A converged science must be followed with converged policy and action. Accordingly, the Panel must be co-embedded with a second mechanism, either the Intergovernmental Forum or the Standard Setting Commission. In so doing, these mechanisms’ strengths overcome the other’s limitations. Where the Forum and Commission lack scientific credibility, the Independent Panel strengthens it. The latter’s scientific assessments could inform the Commission’s or the Forum’s proceedings, thus strengthening the science-policy interface, and generating more informed policy debate. Meanwhile, the Forum and Commission enable the Independent Panel’s findings to translate into tangible action.

Paired with the Independent Panel, the Intergovernmental Forum has the potential to be highly impactful. Drawing on the evidence base generated by the Panel, it would engender policy convergence for all types of action. Offering a space for member state discussion, consultation, and coordination is “essential for managing the political dimension that inevitably characterizes an international crisis,”^87^ and can bring about improved effectiveness and greater harmonization.^88^ The Forum would bring about greater democratic and political legitimacy, building trust and accountability between actors, shaping consensus, and, ideally, bringing about better and more equitable solutions through meaningful and representative participation.

Alternatively, the integration of a Standard Setting Commission could strongly promote harmonized, tangible, and technocratic action that is direly needed across One Health. Setting standards referenced by the instrament creates pressure to comply, such that the Commission’s outputs could become nearly compulsory, thus bringing about greater progress and compliance with instrament obligations. This mechanism can also serve countries with less capacity to comply with instrament obligations, by facilitating monitoring and targeted capacity-building.

## Conclusion

Pandemic threats, whether of zoonotic origin or as a result of drug resistance, are quintessential One Health issues.^89^ Coordination across the human-animal-environment interface is a challenge at all levels of governance, but any effort to prevent, prepare, and eventually combat future pandemics is hopeless without effective multisectoral engagement. Now is the time to forge and deepen partnerships and collaborations so that we may prevent the world from future health threats, and so that when emergencies do arise, the global governance system is empowered with a shared mission, clear mandates, and has the capacity to manage even the most unexpected of threats.^90^

## Note

This work was supported by the Canadian Institutes of Health Research [#149542], the Social Sciences & Humanities Research Council [#895-2022-1015], and the Wellcome Trust [222422/Z/21/Z]. The funding bodies were not involved in the study design, data collection, analysis, interpretation or writing.
